# Editorial: Biology of Stress Granules in Plants

**DOI:** 10.3389/fpls.2022.938654

**Published:** 2022-06-09

**Authors:** Monika Chodasiewicz, J. C. Jang, Emilio Gutierrez-Beltran

**Affiliations:** ^1^Center for Desert Agriculture, Biological and Environmental Science and Engineering Division (BESE), King Abdullah University of Science and Technology (KAUST), Thuwal, Saudi Arabia; ^2^Department of Horticulture and Crop Science and Department of Molecular Genetics, Center for Applied Plant Sciences and Center for RNA Biology, The Ohio State University, Columbus, OH, United States; ^3^Instituto de Bioquímica Vegetal y Fotosíntesis, Universidad de Sevilla and Consejo Superior de Investigaciones Científicas (CSIC), Seville, Spain; ^4^Departamento de Bioquímica Vegetal y Biología Molecular, Facultad de Biología, Universidad de Sevilla, Sevilla, Spain

**Keywords:** mRNA metabolism, stress signaling, plant stress granules, biomolecular condensates, RNA-binding proteins

## Introduction

Eukaryotic cells have developed sophisticated mechanisms to survive under ever-changing environments which include compartmentalization of translationally arrested mRNA molecules and proteins into a type of membraneless cytoplasmic foci called stress granules (SGs). Stress granules were first identified as phase-dense cytoplasmic particles formed in mammalian cells when subjected to heat shock (Arrigo et al., 1988). To date, intensive studies in yeast and animal model systems have helped elucidate the major molecular composition of SGs (Jain et al., 2016; Markmiller et al., 2018; Marmor-Kollet et al., 2020). SGs are typically consisted of small ribosomal subunits, various translation initiation factors (eIFs), poly(A)-binding proteins (PABs), and a variety of RNA-binding proteins (RBPs) and non-RNA-binding proteins. Although SGs were initially thought to facilitate mRNA translational arrest during stress, it has been well-documented that SGs play a more active role in stress response, mRNA triage and stress signaling, among other processes (Hofmann et al., 2021). The mechanisms governing the assembly of SGs have been recently extensively discussed (Schmit et al., 2021). Growing evidence have now suggested that SGs can be classified as droplets formed by liquid-liquid phase separation (LLPS) in the cytoplasm (Jain et al., 2016; Yang et al., 2020).

In contrast to mammalian or yeast model system, research in the plant SGs field is still in its infancy. Despite very recent works that have begun to provide a better understanding on some of the mechanistic questions, the investigation of plant SGs still represents an emerging field. Therefore, numerous knowledge gaps remain to be filled. Here, we share with the plant biology community a Research Topic that aims to highlight the most current findings in the field of SG biology in plants.

## Novel Advances in Plant Stress Granules

It is well-known that stress granule assembly takes place in the cytoplasm (Hofmann et al., 2021). However, several recent reports have pointed to the existence of SG-like foci in the chloroplasts. William Zerges's lab was the first to identify plastidial RNA granules, using the unicellular green algae model organism *Chlamydomonas rehinharti* (Uniacke and Zerges, 2008). In this Research Topic, Chodasiewicz et al. demonstrated that plastidial SGs (cpSGs) were also found in the chloroplasts of higher plants. In this study, scientists demonstrated that *Arabidopsis thaliana* (Arabidopsis) cpSGs shared similar properties with cytoplasmic SGs, including the dynamic of assembly/disassembly and sublayer composition. Consistent with previous findings in cytoplasmic SGs (Gutierrez-Beltran et al., 2015; Kosmacz et al., 2019; Kosmacz and Skirycz, 2020), heat-induces cpSGs (purified using SNOWY COTYLEDON 1—GFP as bait) also contained mRNA molecules, metabolites, and proteins. A proteomic study revealed the presence of proteins traditionally linked with cytoplasmic SG assembly and function, pointing toward that the composition between the two classes of SGs is rather conserved. In addition to composition conservation, a large fraction of the cpSGs proteome corresponded to stress-related proteome suggesting that, similarly to cytoplasmic SGs, plastidial SGs might also play a key role in protecting proteins from unfolding and aggregation during stress. Interestingly, stress-related metabolites such proline was identified in both cytosolic SGs and cpSGs, further supporting the metabolite conservation in granule composition. These results suggest that metabolites, in addition to key protein factors, might be required for SGs formation/disassembly (Kosmacz et al., 2018).

While much attention has been paid to SG assembly, the disassembly mechanisms are still less understood. Autophagy is a catabolic process that removes damaged organelles or cytoplasmic components and often relies on the formation of membrane-bound autophagosomes (Mizushima, 2009). In animal and yeast model systems, autophagy controls SGs disassembly through a process known as granulophagy (Buchan et al., 2013; Mahboubi and Stochaj, 2017; Hofmann et al., 2021). Whether this process could occur in plants is still to be investigated. In this Research Topic, Field et al. reported that Arabidopsis SUPPRESSOR OF GENE SILENCING 3 (SGS3)-containing bodies could be degraded by autophagy during extended hypoxia in a process dependent on the calcium-sensor protein CALMODULIN-LIKE 38 (CML38). Although SGS3 was previously characterized as a component of siRNA bodies (Jouannet et al., 2012), both CML38 and SGS3 proteins could co-localize with the SG marker RBP47 in *Nicotiana benthamiana*. The RNA-binding protein SGS3 was identified as a direct interacting partner of CML38 in a Bacterial Adenylate Cyclase-based Two-Hybrid (BACTH) assay. In addition to SGS3, CELL DIVISION CYCLE 48 (CDC48) and DUF5811-5 were also identified as CML38 interacting partners. Remarkably, the AAA+-ATPase CDC48 was previously found to facilitate granulophagy in yeast (Buchan et al., 2013). With those results, Field et al. revealed that CML38-dependent autophagy of SGS3 bodies in Arabidopsis also involves CDC48. Taken together, the authors proposed a model in which both CDC48 and CML38 coordinate SGS3 body granulophagy in response to hypoxia stress in plants.

In plants, like in other organisms, stress-activated signaling pathways trigger the assembly/disassembly of SGs, but what are the required factors for SG assembly/disassembly, and ultimately the SG function remain unknown. More importantly, it is still unclear to what extent SGs can contribute to plant stress responses (Kosmacz et al., 2018; Chodasiewicz et al., 2020; Jang et al., 2020; Gutierrez-Beltran et al., 2021). In this Research Topic, in a comprehensive review, Maruri-López et al. reported more recent advances in SG composition, organization, dynamics, regulation, and their relationship to other cytoplasmic granules, including processing bodies. The review also comprehensively linked the most exciting findings of SG research from mammalian/yeast with the discoveries in plants. A model for stress SG assembly and disassembly was proposed based on the accumulated evidence from plant and non-plant models. Comparative view of SG biology across different kingdoms provides excellent source of information and advancement to the SG research field.

## Rna-Binding Proteins, Past, Present, and Future

One of the most intensively studied proteins in the mammalian SG field is RAS GTPASE-ACTIVATING PROTEIN-BINDING PROTEIN (G3BP), which acts as a key molecular switch in regulating RNA-dependent SG assembly (Guillen-Boixet et al., 2020; Yang et al., 2020). Whereas there are only two *G3BP* genes in mammals (*G3BP1* and *G3BP2*), there are nine genes in Arabidopsis. This makes the characterization of *G3BPs* in plants very challenging. In this Research Topic, Abulfaraj et al. summarize the recent findings of the dynamics and role of G3BPs in plants. In this very timely review, a large part of the article is focused on understanding the possible roles of plant G3BP proteins in SG function. Although several members of the plant G3BP family have been observed to localize in cytoplasmic foci under stress, only the G3BP7 isoform was co-localized with a SG marker (Krapp et al., 2017; Reuper et al., 2021). Whether any of the plant G3BPs is required for SG assembly, similarly to its mammalian homologs, is still an open question. However, it seems to be unlikely due to potential functional redundancy of the large G3BP family in plants.

Although the exact functions have not been completely elucidated, the RNA-binding proteins (RBPs) are the key components of SGs (Kosmacz et al., 2018; Gutierrez-Beltran et al., 2021). RBPs are required for RNA metabolism in both nucleus and cytoplasm, and they have been reported to be crucial for stress adaptation (Chantarachot and Bailey-Serres, 2018). Despite this fundamental importance, our understanding of plant RBPs is rudimentary. In a comprehensive review in this Research Topic, Yan et al. summarizes the recent findings in plant RBPs, linking SG assembly and ABA signaling with plant stress responses. A model depicting the regulatory functions of plant RBPs in various cellular compartments including SGs, in adaptation to abiotic stress was presented. It was proposed that, under stress condition, RBPs may regulate gene expression in an ABA-dependent or independent manner and control the response by modulating transcriptional or posttranscriptional mechanisms.

## Author Contributions

The authors jointly defined the content of this Research Topic and all participated in the editing process. All authors listed have made a substantial, direct, and intellectual contribution to the work and approved it for publication.

## Funding

Research of MC lab was supported by King Abdullah University for Science and Technology (KAUST) in Saudi Arabia. JJ lab research was supported by the USA National Science Foundation (MCB-1906060) and the Ohio Agricultural Research and Development Center grant (OHOA1627). Research of EG-B lab was supported by the Spanish Grant PID2020-119737GA-I00 funded by MCIN/AEI/ 10.13039/501100011033.

## Conflict of Interest

The authors declare that the research was conducted in the absence of any commercial or financial relationships that could be construed as a potential conflict of interest.

## Publisher's Note

All claims expressed in this article are solely those of the authors and do not necessarily represent those of their affiliated organizations, or those of the publisher, the editors and the reviewers. Any product that may be evaluated in this article, or claim that may be made by its manufacturer, is not guaranteed or endorsed by the publisher.
